# Higher naloxone dosing in a quantitative systems pharmacology model that predicts naloxone-fentanyl competition at the opioid mu receptor level

**DOI:** 10.1371/journal.pone.0234683

**Published:** 2020-06-16

**Authors:** Ronald B. Moss, Meghan McCabe Pryor, Rebecca Baillie, Katherine Kudrycki, Christina Friedrich, Mike Reed, Dennis J. Carlo

**Affiliations:** 1 Adamis Pharmaceuticals Corp, San Diego, CA, United States of America; 2 Rosa & Co. LLC, San Carlos, CA, United States of America; Universidade Federal do Rio de Janeiro, BRAZIL

## Abstract

Rapid resuscitation of an opioid overdose with naloxone, an opioid antagonist, is critical. We developed an opioid receptor quantitative systems pharmacology (QSP) model for evaluation of naloxone dosing. In this model we examined three opioid exposure levels that have been reported in the literature (25 ng/ml, 50 ng/ml, and 75 ng/ml of fentanyl). The model predicted naloxone-fentanyl interaction at the mu opioid receptor over a range of three naloxone doses. For a 2 mg intramuscular (IM) dose of naloxone at lower fentanyl exposure levels (25 ng/ml and 50 ng/ml), the time to decreasing mu receptor occupancy by fentanyl to 50% was 3 and 10 minutes, respectively. However, at a higher fentanyl exposure level (75 ng/ml), a dose of 2 mg IM of the naloxone failed to reduce mu receptor occupancy by fentanyl to 50%. In contrast, naloxone doses of 5 mg and 10 mg IM reduced mu receptor occupancy by fentanyl to 50% in 5.5 and 4 minutes respectively. These results suggest that the current doses of naloxone (2 mg IM or 4 mg intranasal (IN)) may be inadequate for rapid reversal of toxicity due to fentanyl exposure and that increasing the dose of naloxone is likely to improve outcomes.

## Background

Data from the Centers for Disease Control (CDC) has identified a rise of almost 10% in deaths due to drug overdoses killing approximately 71,000 Americans in 2017 [1]. New provisional data from the CDC suggests a drop in overall deaths (68,000) due to drug overdose in 2018 [2]. However, the number of deaths due to illicitly manufactured synthetic opioids, such as fentanyl, continues to rise [2]. Fentanyl is a synthetic opiate that is considered 50 to 100-fold more potent than morphine [3] and like other opiates, binds to the mu opiate receptors in the central nervous system (CNS). Fentanyl is known to cause respiratory depression within minutes of exposure [4, 5]. Rapid brain hypoxia and ultimately death can occur within minutes after fentanyl exposure [6]. The potency of fentanyl is thought to be due to rapid binding and high occupancy of the opiate mu receptors in the brainstem respiratory centers of the CNS [7, 8]. Recent reported overdose systemic blood levels of fentanyl vary widely from to 0.5 ng/ml to 162 ng/ml [9–12]. The mean level of fentanyl exposure was 52.9 ng/ml in one series of overdose patients [13].

Naloxone is a synthetic derivative of oxymorphone that antagonizes opioids and is used as a countermeasure in acute opioid overdose. It has been postulated to antagonize the three opiate receptors in the brain (mu, kappa, alpha) [14]. The current approved doses of naloxone for home or self-administration are 4 mg IN and 2 mg IM. These two different dosage forms approximate similar systemic exposure as the IN administration route results in approximately 45% bioavailability compared to IM [15]. Prior to the new synthetic opioid era, community programs reported nearly 100% naloxone post administration survival rates with approved doses of naloxone [16]. However, the continuing rise of the more potent illicitly manufactured fentanyl and other synthetic opioids has created new challenges for the adequate treatment of overdoses. Naloxone is considered the recommended treatment for acute opioid toxicity [17] but an assessment of adequacy of the current doses has yet to be examined because an assessment in clinical trials would be logistically and ethically problematic.

We developed an opioid receptor QSP model and conducted simulation experiments to evaluate the effects of naloxone dosing. The model examined reported ranges of fentanyl systemic exposure in overdoses and predicted naloxone-fentanyl competition at the mu opioid receptor over a range of naloxone doses. Outcompeting fentanyl with naloxone at the mu opioid receptor in the CNS is critical to reversing opioid toxicity. The simulations examined the change in % mu receptor occupancy by fentanyl after different doses of naloxone administration. In addition, we examined time to a reduction to 50% mu receptor occupancy by fentanyl, as this endpoint has been associated with clinical reversal of opiate toxicity [18].

The results of the simulations suggest that the current approved doses of naloxone (2 mg IM or 4 mg IN) may be inadequate for rapid reversal of toxicity at fentanyl levels observed in overdose victims and that outcomes could be improved with increased doses.

## Methods

**Fig 1:**
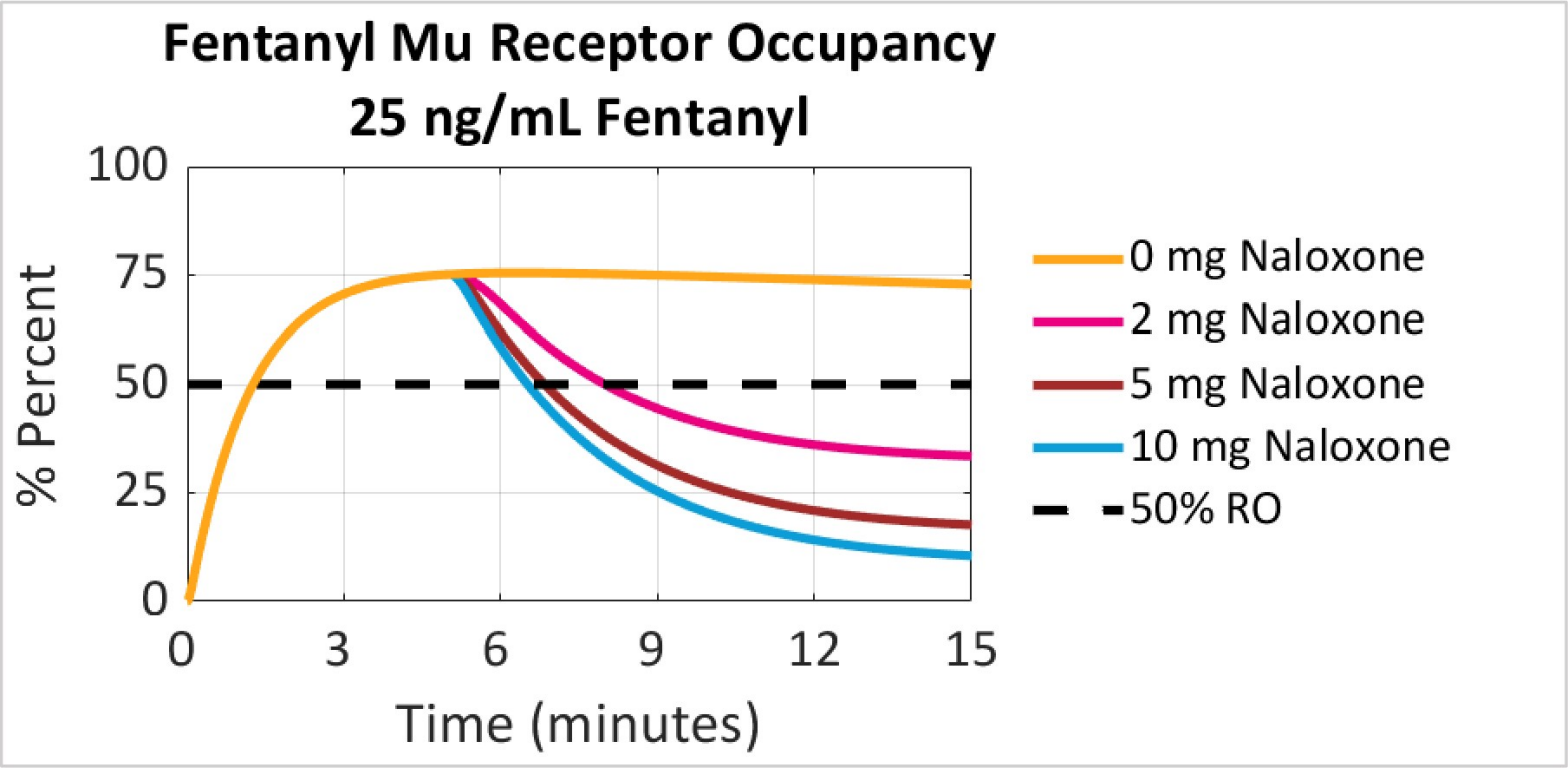
Graphical depiction of the mu receptor submodel. The model accounts for mu receptor synthesis and degradation, competitive binding to the receptor, internalization, recycling, and clearance.

A PhysioPD Research Platform (model) was developed using SimBiology (The MathWorks, MATLAB 2018b). The model includes non-linear ordinary differential equations (ODEs) to represent plasma and brain pharmacokinetics (PK) for fentanyl and naloxone, and mu receptor dynamics. The mu receptor submodel includes standard competitive binding dynamics and accounts for receptor synthesis, internalization, and recycling. (Fig 1 and S1 Table) Parameters to characterize these dynamics were taken from literature and are summarized in Table 1. Plasma PK for IM naloxone was based on a published model [19]. Various routes of administration are possible for fentanyl. Model simulations do not specify a particular route of fentanyl administration, but instead start with a high plasma concentration relevant for overdose scenarios and capture the clearance phase of fentanyl PK. Because of non-specific binding, concentrations in the brain of fentanyl available for binding to mu receptor are difficult to measure experimentally. Published data for the relationship between naloxone dose and mu receptor occupancy [20], and between fentanyl dose, receptor occupancy, and pain relief [21–23] were used to infer brain exposures. For example, a fentanyl plasma level of 5 ng/ml is associated with pain relief [24], and pain relief is evident at mu receptor occupancy above 10% [25]. The model assumes 15% mu receptor occupancy at a plasma concentration of 5 ng/ml. Using a Ki of 1.35 nM (Table 1), this suggests a fentanyl concentration at the mu receptor of ~0.24 nM for pain relief. The model contains a single subject representing a habitual opioid user. The amount of mu receptor and rate of receptor internalization have been set to represent a subject with opioid tolerance [26, 27].

**Table 1:** Data from the literature on receptor dynamics used in the model *.

Species	Parameter	Model Value	Range in literature	References
Naloxone	Mu receptor binding Ki	1.115 nM	0.16–6.6 nM	[29, 35–50]
Fentanyl	Mu receptor binding Ki	1.35 nM	0.76–3 nM	[41, 43, 49, 51–53]
Mu Receptor	Number of receptors in brain	2.37 x10^7^ fmol/brain	1.9x10^7^–3x10^7^ fmol/brain	[45, 54–57]
Mu Receptor	Unbound receptor half-life	12 hours	12 hours	[58]
Mu Receptor	Bound receptor half-life	7 hours	7 hours	[58]
Mu Receptor	Naloxone-bound receptor internalized after 30 minutes	15%	Little to none	[59, 60]
Mu Receptor	Fentanyl-bound receptor internalized after 30 minutes	80%	50–88%	[61]
Naloxone	Plasma PK	Based on published model		[19]
Fentanyl	Plasma PK	Based on published models		[10, 11, 62–70]
Fentanyl	Plasma concentration, surgery	10	1–20 ng/mL	[71–82]
Fentanyl	Plasma concentration, loss of consciousness	25	3–34	[68, 83–86]
Fentanyl	Plasma concentration in overdose	50	1–102	[10, 11, 62–69]
Fentanyl	Appearance in the brain	<1 minute post dose	<1 minute post dose	Based on appearance rates [42, 87–89]
Naloxone	Appearance in the brain	<1 minute post dose	<1 minute post dose	[20, 28, 90, 91]
Fentanyl	Brain concentration	Based on dose	Based on dose	[87, 92–95]
Naloxone	Brain concentration	Based on dose	Based on dose	[20]

Model simulations illustrate the time course of fentanyl concentration in plasma and brain, naloxone concentration in plasma and brain, and fentanyl and naloxone mu receptor occupancy. We assumed plasma exposure level of fentanyl at 50 ng/ml as mid-range exposure based on plasma concentrations in overdose victims (Table 1) and also examined fentanyl exposure levels of 25 ng/ml and 75 ng/ml. Naloxone doses examined were 2 mg, 5 mg, and 10 mg IM. Naloxone administration was assumed to occur as a single dose at 5 minutes after peak fentanyl concentration in plasma, which may reasonably reflect response time following initial symptoms of overdose.

## Results

A single dose of IM naloxone was administered at dose levels of 2 mg, 5 mg, and 10 mg IM five minutes after peak fentanyl plasma concentration of 25 ng/mL, 50 ng/mL or 75 ng/mL.

**Fig 2:**
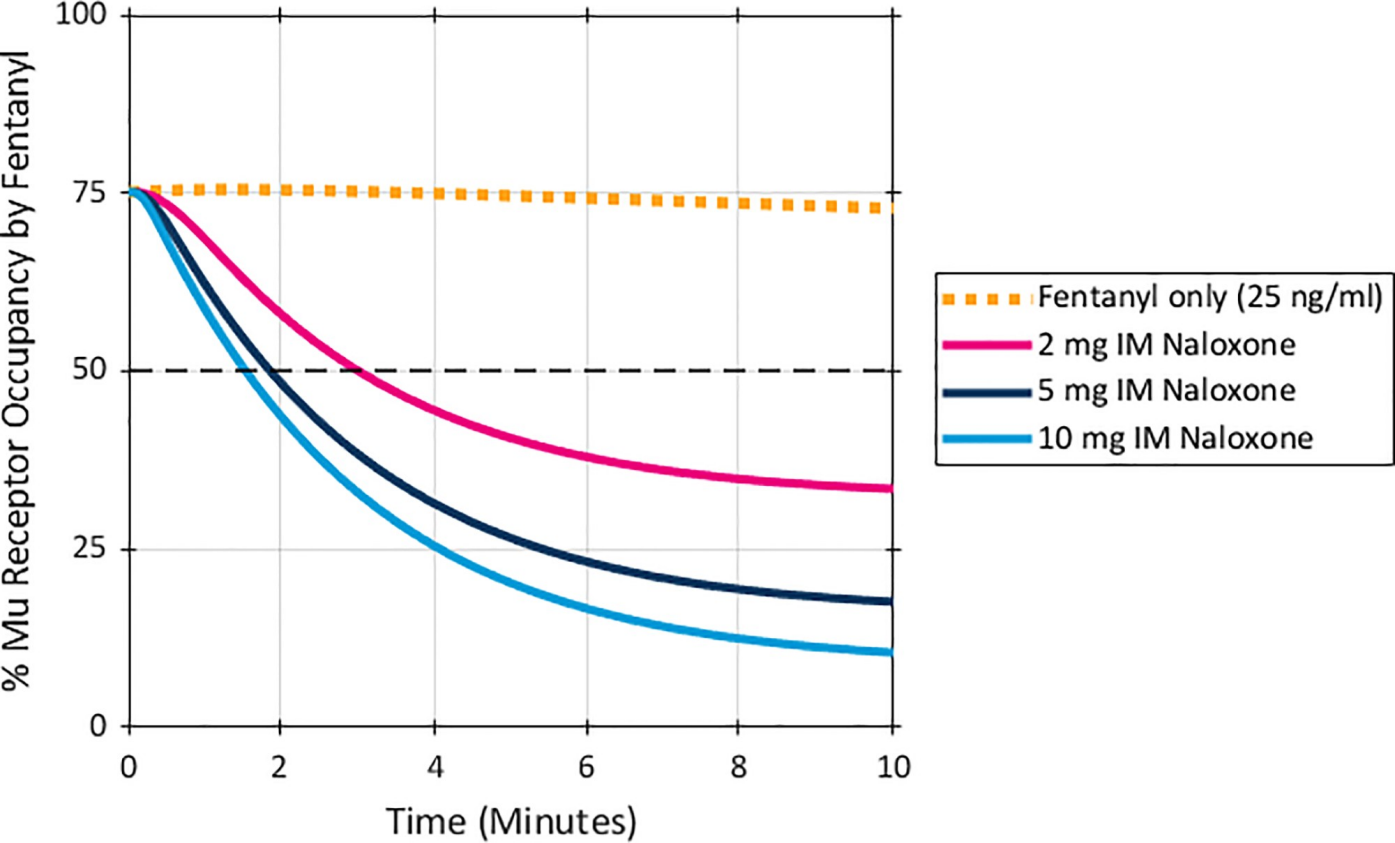
Predicted % mu receptor occupancy by fentanyl after a peak fentanyl plasma concentration of 25 ng/ml. Naloxone was given at time 0 and simulated based on an IM dose PK. Dashed black line marked at 50% receptor occupancy.

As noted in Fig 2, model simulations predicted that a fentanyl overdose exposure level of 25 ng/ml leads to 73% mu receptor occupancy by fentanyl without competition from naloxone. Administration of naloxone leads to rapid reduction in mu receptor occupancy by fentanyl due to competition by naloxone, as would be expected based on naloxone’s fast appearance in brain [20, 28], and the rapid dissociation rates of fentanyl and naloxone [29]. After administering a 2 mg IM of naloxone, mu receptor occupancy by fentanyl was predicted to decrease to 33% within ten minutes of naloxone being administered. The model also predicted that giving a 5 mg IM dose of naloxone would decrease mu receptor occupancy by fentanyl to 17% and giving a 10 mg IM dose of naloxone would decrease of mu receptor occupancy by fentanyl to 9% within ten minutes.

The time to decreasing mu receptor occupancy by fentanyl to below 50% with exposure level of fentanyl at 25 ng/ml with various naloxone doses was also examined. While the receptor occupancy associated with respiratory arrest may vary across patients, it is nonetheless instructive to observe the effect of dose escalation on the time to cross a given level of receptor occupancy. As shown in Fig 2, increasing dose is associated with a faster time to cross the threshold. A dose of 2 mg IM reduced mu receptor occupancy by fentanyl to 50% in 3 minutes, while doses of 5 mg IM and 10 mg IM of naloxone resulted in a decrease to 50% mu receptor occupancy by fentanyl in 2 and 1.5 minutes, respectively.

**Fig 3:**
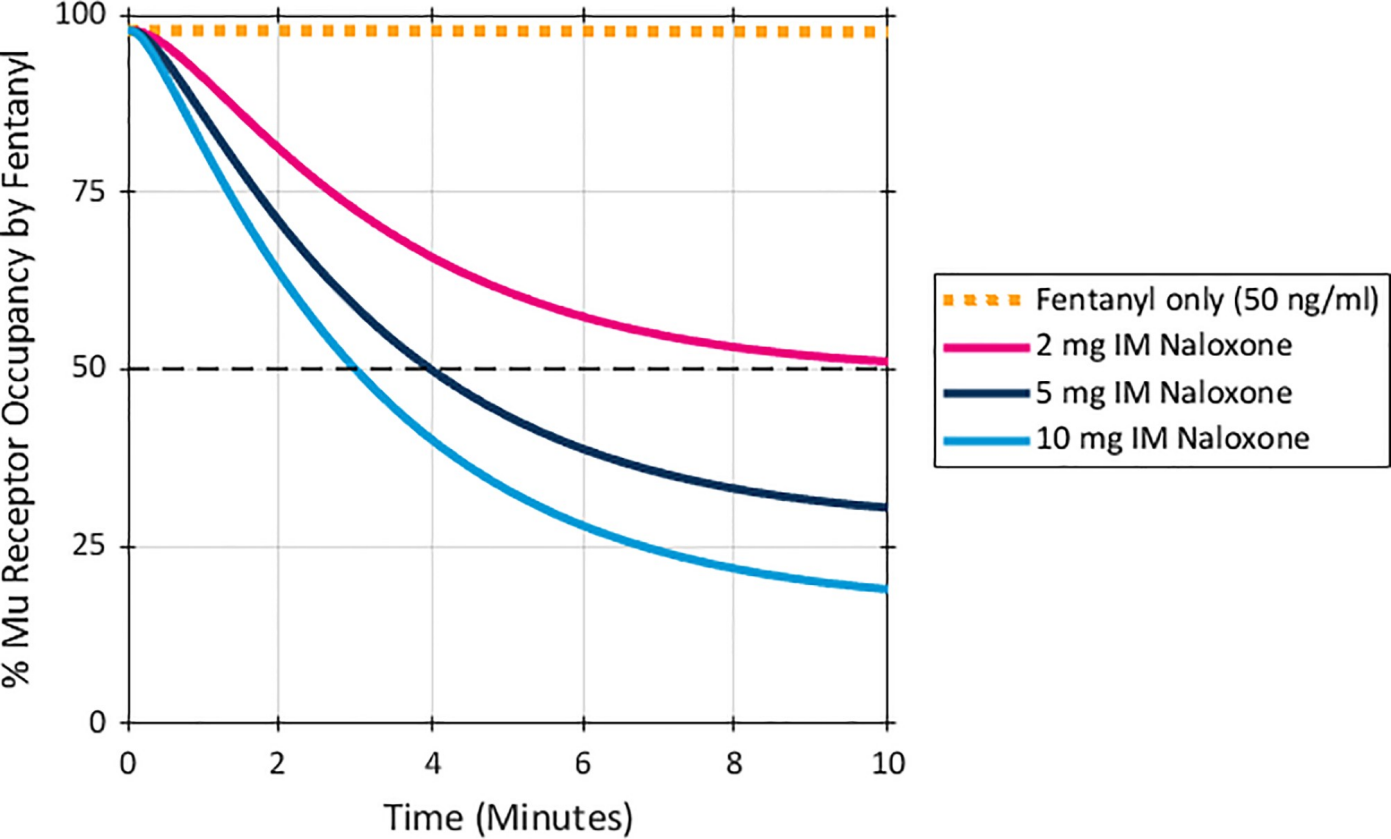
Predicted %mu receptor occupancy by fentanyl after a peak fentanyl plasma concentration of 50 ng/ml. Naloxone was given at time 0 and simulated based on an IM dose PK. Dashed black line marked at 50% receptor occupancy.

The next set of simulations examined a higher fentanyl plasma exposure of 50 ng/ml which was predicted to result in 97% mu receptor occupancy by fentanyl without naloxone administration, as shown in Fig 3. At this fentanyl exposure level, a dose of 2 mg IM naloxone was predicted to decrease mu receptor occupancy by fentanyl to 50%, while naloxone doses of 5 mg and 10 mg IM reduced mu receptor occupancy by fentanyl to 29% and 17%, respectively.

The time to 50% mu receptor occupancy by fentanyl with an exposure to fentanyl level of 50 ng/ml again showed a significant dose effect. As shown in Fig 3, a dose of 2 mg IM of naloxone reduced mu receptor occupancy by fentanyl to 50% in 10 minutes, while the 5 mg and 10 mg IM doses of naloxone shortened the time to a 50% decrease in mu receptor occupancy by fentanyl to 4 minutes and 3 minutes, respectively.

**Fig 4:**
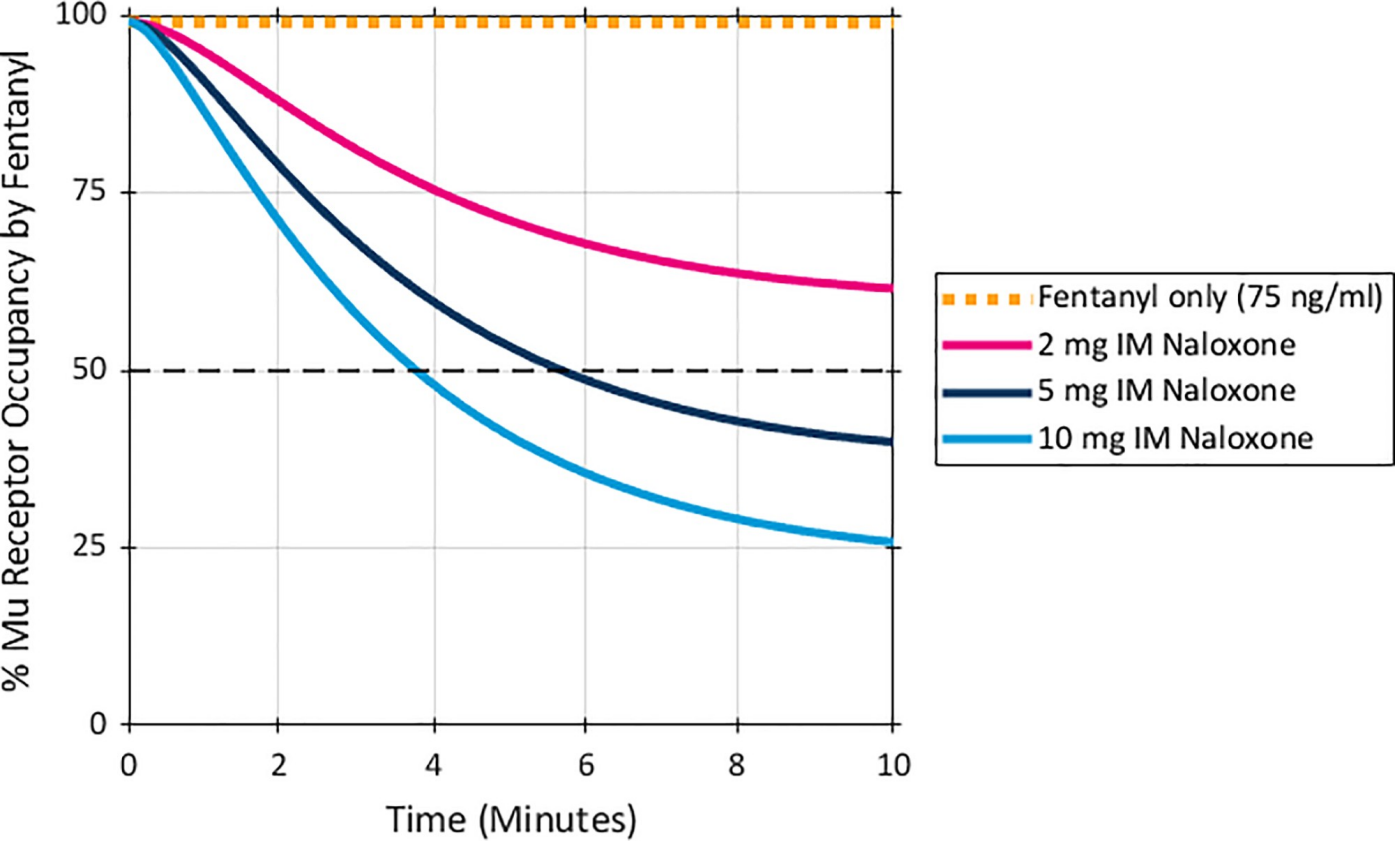
Predicted % mu receptor occupancy by fentanyl after a peak fentanyl plasma concentration of 75ng/ml. Naloxone was given at time 0 and simulated based on an IM dose PK. Dashed black line marked at 50% receptor occupancy.

Results for the even higher fentanyl exposure levels (75 ng/ml), as shown in Fig 4, were consistent with the patterns observed at the lower exposures. The model predicted a 98.9% mu receptor occupancy by fentanyl without naloxone administration. A dose of 2 mg IM, naloxone reduced mu receptor occupancy by fentanyl to 62% at 10 minutes after naloxone administration, while naloxone doses of 5 mg and 10 mg IM decreased receptor occupancy by fentanyl to 40% and 26%, respectively, ten minutes post administration.

As was the case at the lower fentanyl exposures, the time to decreasing to 50% mu receptor occupancy by fentanyl after an exposure level of 75 ng/ml was responsive to dose. As shown in Fig 3, a dose of 2 mg IM did not reduce mu receptor occupancy by fentanyl to 50% within 10 minutes at this exposure level, while doses of 5 mg and 10 mg IM of naloxone reduced mu receptor occupancy by fentanyl below 50% in 5.5 minutes and 4 minutes, respectively.

## Discussion

Rapidly decreasing opioid mu receptor occupancy seems imperative for successful clinical reversal, particularly for the more potent synthetic opioids. Sommerville, et al., reported [30] a study where 83% of patients required greater than 2 mg naloxone doses prior to a clinical response. Of particular concern is that 36% of fatal deaths had a drug overdose within seconds to minutes after drug use. Upon Emergency Medical Service (EMS) arrival, 90% were pulseless. This report and others support the notion that acutely administered higher doses of naloxone are needed for rapid and adequate clinical reversal, particularly when the more potent synthetic opioids are abused.

Using an opioid receptor QSP model we predicted naloxone-fentanyl interaction at the mu opioid receptor over a range of naloxone doses. We examined three levels of fentanyl exposure in the plasma found in overdose patients. The high receptor mu occupancy of fentanyl at the doses observed in this model without naloxone administration is consistent with reports of the potency of this synthetic opioid and its relation to a recent spike in overdoses and deaths. Fentanyl is known to be persistently lipophilic and has been described as having a rapid transport in and out of the CNS [31].

The results of simulations in our model suggest that at exposure levels of fentanyl capable of resulting in respiratory depression and death (50 ng/ml and 75 ng/ml), the current doses of naloxone (2 mg IM and 4 mg IN) may be inadequate for a rapid successful reversal. Respiratory depression occurs within minutes of fentanyl exposure [4] and brain damage and death occur within 6 minutes of anoxia [32]. With a fentanyl exposure level of 50 ng/ml, the model results suggested that the time to 50% fentanyl receptor occupancy for 2 mg IM of naloxone was 10 minutes. Reversing fentanyl toxicity in 10 minutes is likely too long for a successful reversal. In contrast, the time to 50% fentanyl occupancy was 4 and 3 minutes, for 5 mg and 10 mg of naloxone IM administrations, respectively. Even more concerning was the finding from the model that at higher fentanyl exposure levels (75 ng/ml), a 2 mg IM of naloxone did not result in decreasing mu receptor occupancy by fentanyl below 50% within ten minutes after administration. Higher doses of naloxone, 5 mg and 10 mg IM, however, resulted in time to decreasing to 50% mu receptor occupancy by fentanyl in 5.5 and 4 minutes, respectively. These response times are still within a window for a potential successful resuscitation.

The model results also suggested that at lower levels of fentanyl exposure (25 ng/ml), current doses of naloxone (2 mg IM or 4 mg IN) may be adequate, with a relatively short time to 50% occupancy displacement of fentanyl (3 minutes). However, the assumption of this model is that naloxone would be given after 5 minutes of fentanyl exposure. Giving a higher dose of naloxone would still be advantageous if naloxone administration was delayed for longer than 5 minutes after drug exposure. In addition, it is impractical to ascertain the level of fentanyl exposure in an overdosed patient. Therefore, undertreating the patient, with the current doses of naloxone, based on model predictions, may have dire consequences.

The model uses a number of assumptions but is consistent with other studies suggesting that the mu receptor occupancy by fentanyl increases with an increase in systemic fentanyl exposure [21], and that naloxone receptor occupancy increases as naloxone dose increases [23].

Our model has a number of limitations. It represents plasma and brain PK and mu receptor dynamics based on our current understanding of opioids and the brain, which is incomplete. It uses several plausible assumptions which may not always be valid in actual overdoses. It does not examine implications of different routes of fentanyl administration or of repeat fentanyl dosing. The current model also does not account for variability between patients and only examines one patient type, a habitual opioid user. Nonetheless, in the absence of definitive data and in light of the practical impossibility of a controlled clinical trial, the model vastly improves upon qualitative statements and mental interpolations by utilizing and integrating available clinical and mechanistic evidence and known receptor competition dynamics. The results of the model simulations suggesting that at lower exposure levels of fentanyl, current doses of naloxone may be adequate for reversal of opioid toxicity are consistent with clinical observations. The simulation results suggesting that current doses of naloxone are inadequate for higher doses of fentanyl exposure (50 ng/ml and 75 ng/ml) are also consistent with clinical results. The model demonstrates that all of these observations are consistent with a receptor competition mechanism and helps quantify the additional benefit of the higher 5 mg and 10 mg IM doses. These analyses strongly support the idea that higher doses of naloxone (5 mg IM and 10 mg IM), as examined in this model would appear to be superior compared to the current naloxone doses (2 mg IM or 4 mg IN), in many overdose situations.

## Conclusions

In summary, this study examined a simulation model of mu receptor occupancy and supports the notion that naloxone may be underdosed in many situations of fentanyl exposure. The risk associated with underdosing of naloxone is an unsuccessful reversal of opioid toxicity leading to death, which outweighs the risk of opioid withdrawal [33]. This model supports the notion that higher doses of naloxone are required as a countermeasure to the new synthetic opioid epidemic [34].

## Supporting information

S1 Fig Simulated naloxone receptor occupancy vs. peak plasma concentration is consistent with published data from Johansson et al. (Johansson et al. 2019). Simulated naloxone receptor occupancy reflects binding affinity and interstitial fluid (ISF) concentration. Relevant IM and intranasal (IN) doses are indicated on the figure. IM administration has a higher bioavailability than intranasal (IN) administration, allowing for a higher plasma concentration at the same dose.
(TIF)

S2 Fig (TIFF)

S1 Table (TIF)
