# Correction: Higher naloxone dosing in a quantitative systems pharmacology model that predicts naloxone-fentanyl competition at the opioid mu receptor level

**DOI:** 10.1371/journal.pone.0240148

**Published:** 2020-09-29

**Authors:** The PLOS ONE Staff

Fig 1 is incorrect. The publisher apologizes for the error. Please see the correct Fig 1 here.

**Fig 1:**
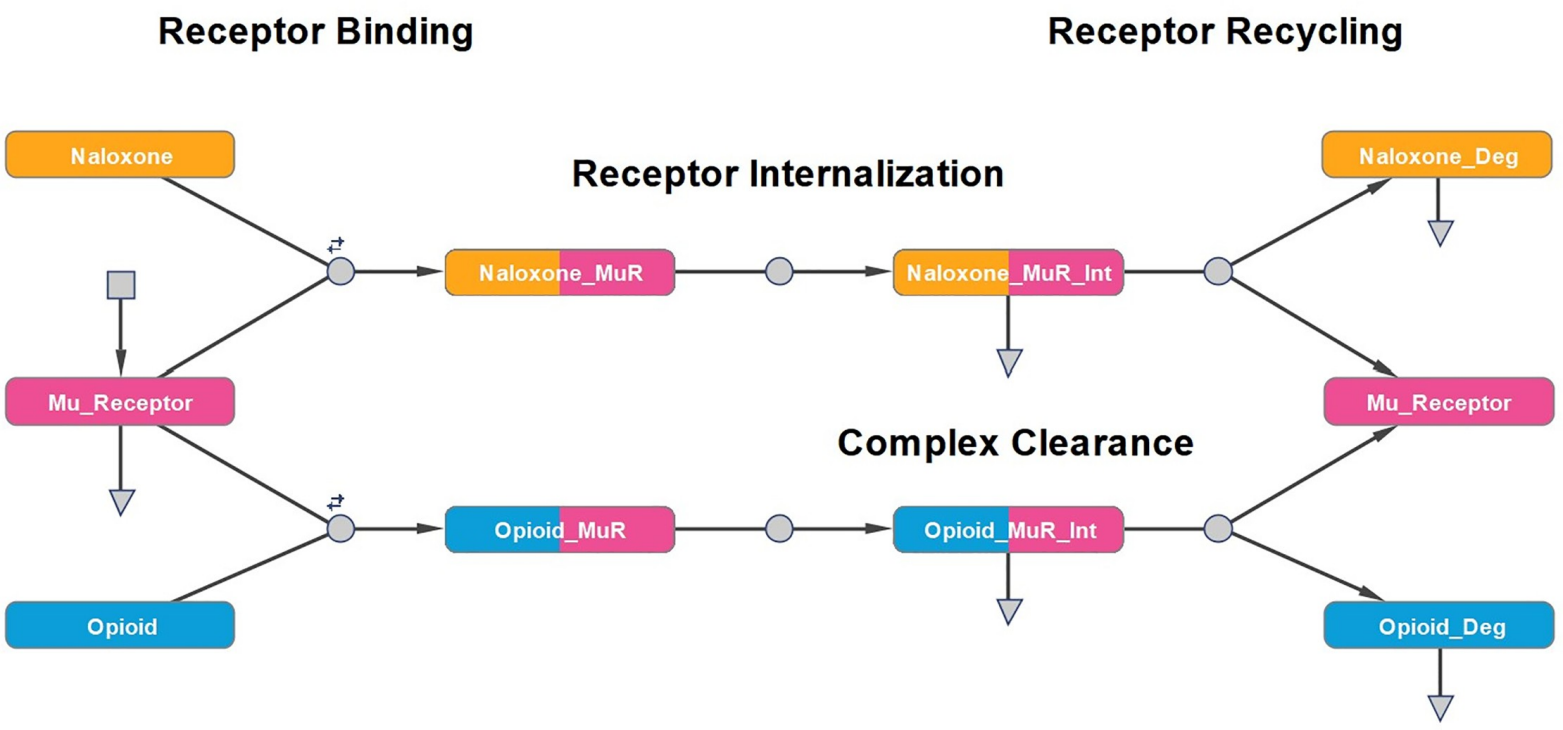
Graphical depiction of the mu receptor submodel. The model accounts for mu receptor synthesis and degradation, competitive binding to the receptor, internalization, recycling, and clearance.
