# Supplementary figures and images for: Higher naloxone dosing in a quantitative systems pharmacology model that predicts naloxone-fentanyl competition at the opioid mu receptor level
Source: PLoS One. 2020 Jun 16;15(6):e0234683. doi: 10.1371/journal.pone.0234683 (PMC7297366; doi:10.1371/journal.pone.0234683)

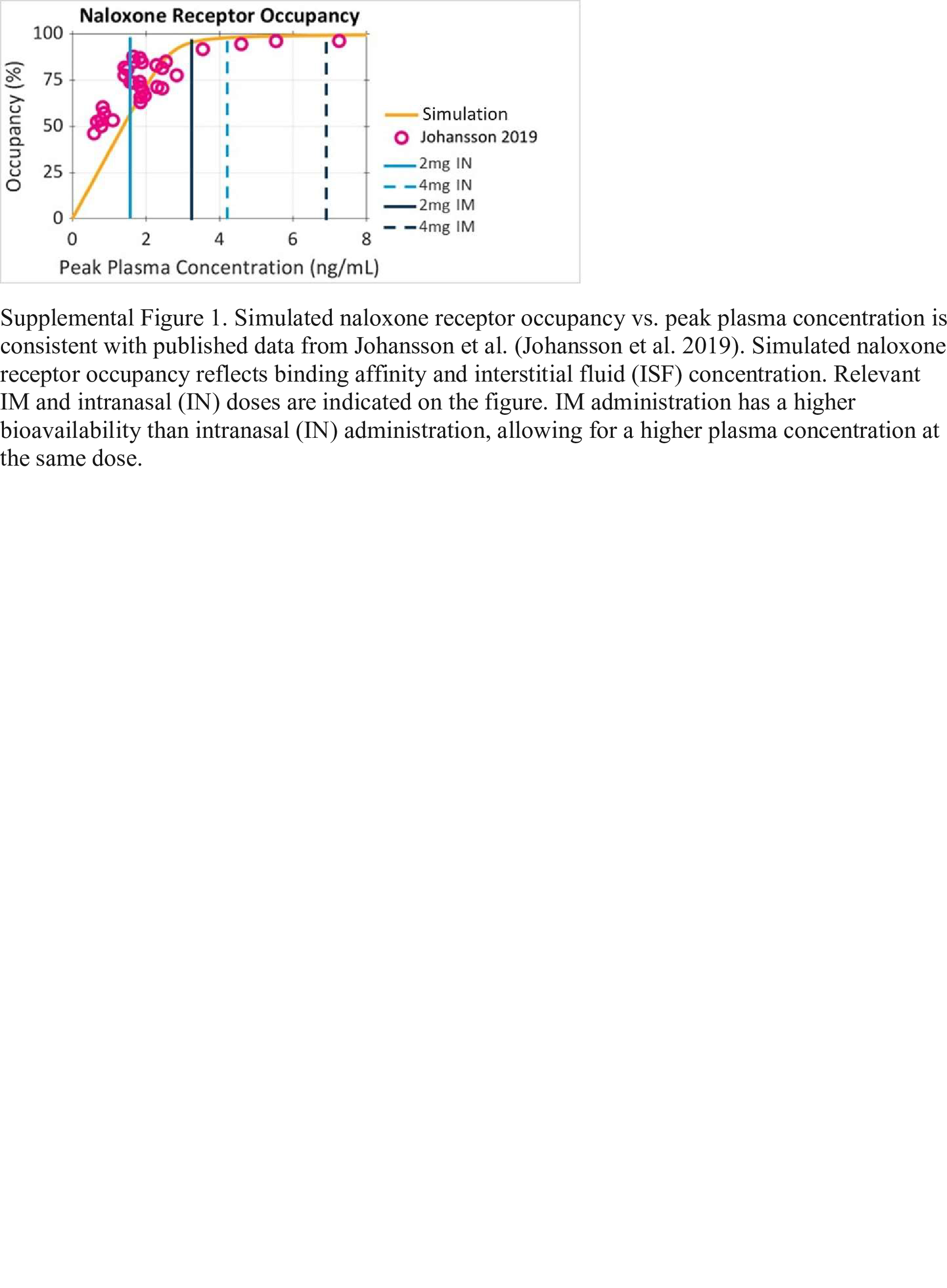

Supplement: S1 Fig — Simulated naloxone receptor occupancy reflects binding affinity and interstitial fluid (ISF) concentration. Relevant IM and intranasal (IN) doses are indicated on the figure. IM administration has a higher bioavailability than intranasal (IN) administration, allowing for a higher plasma concentration at the same dose. (TIF) [file pone.0234683.s001.tif]

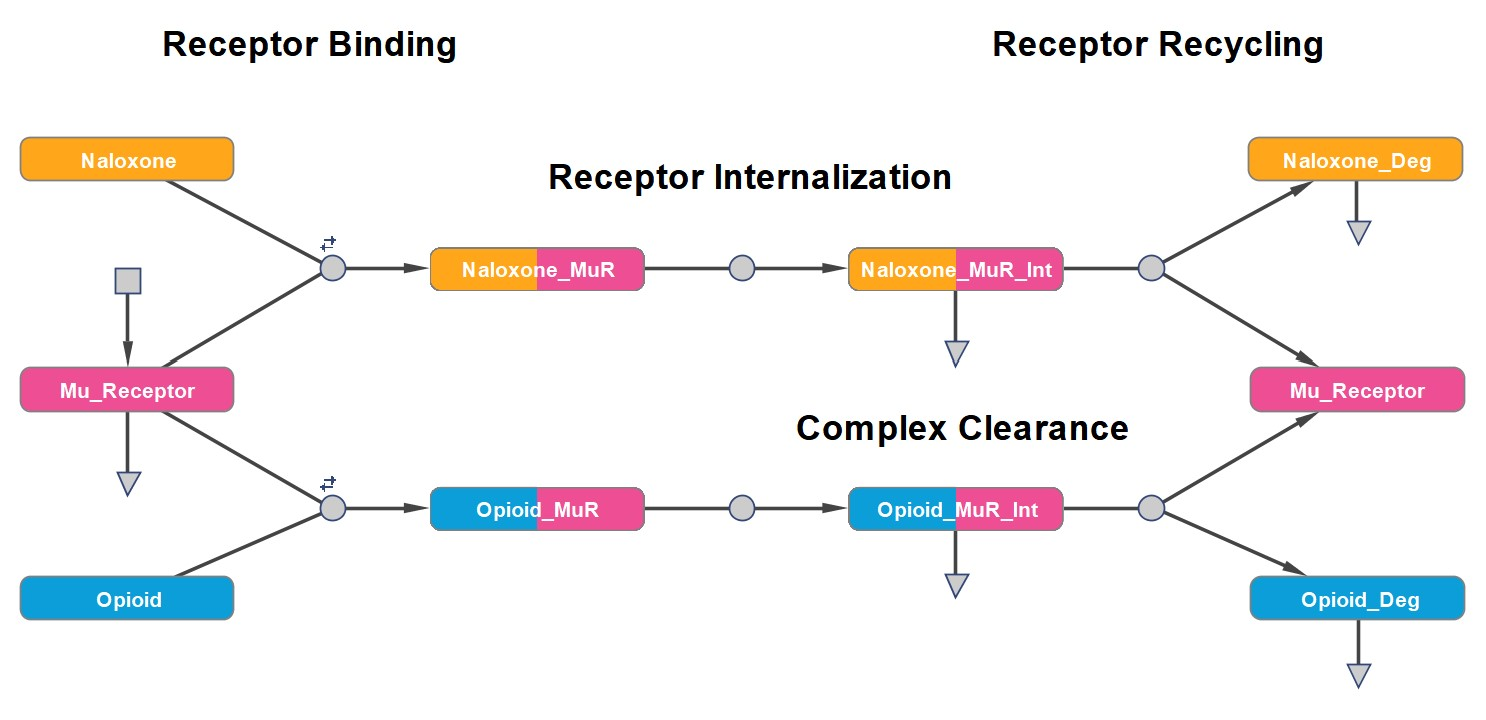

Supplement: S2 Fig — (TIFF) [file pone.0234683.s002.tiff]

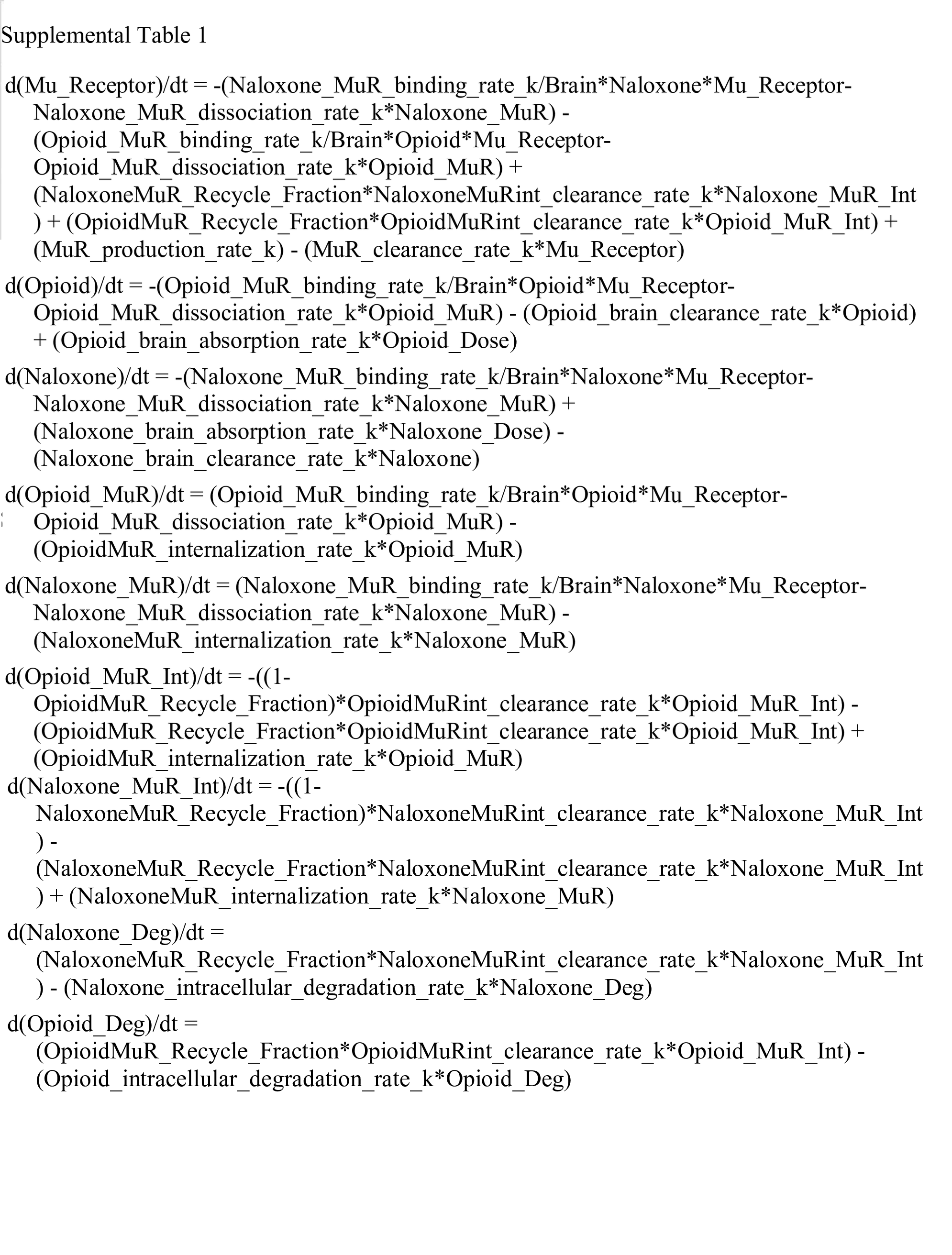

Supplement: S1 Table — (TIF) [file pone.0234683.s003.tif]
